# The Mammalian Phenotype Ontology as a tool for annotating, analyzing and comparing phenotypic information

**DOI:** 10.1186/gb-2004-6-1-r7

**Published:** 2004-12-15

**Authors:** Cynthia L Smith, Carroll-Ann W Goldsmith, Janan T Eppig

**Affiliations:** 1 The Jackson Laboratory, 600 Main Street, Bar Harbor, ME 04609, USA; 2 Massachusetts College of Pharmacy and Health Sciences, School of Pharmacy Manchester, 1260 Elm Street, Manchester, NH 03101, USA

## Abstract

The Mammalian Phenotype (MP) Ontology enables robust annotation of mammalian phenotypes in the context of mutations, quantitative trait loci and strains that are used as models of human biology and disease. The MP Ontology supports different levels and richness of phenotypic knowledge and flexible annotations to individual genotypes. It continues to develop dynamically via collaborative input from research groups, mutagenesis consortia, and biological domain experts. The MP Ontology is currently used by the Mouse Genome Database and Rat Genome Database to represent phenotypic data.

## Abstract 2

The Mammalian Phenotype (MP) Ontology enables robust annotation of mammalian phenotypes in the context of mutations, quantitative trait loci and strains that are used as models of human biology and disease.

## Background

Mammalian phenotypes are complex and the term itself is imprecise. Generally, we use the word phenotype in referring to the appearance or manifestation of a set of traits in an individual that result from the combined action and interaction of genotype and environment.

Because mouse is the premier model organism for the study of human biology and disease, the goal of comparative phenotyping and building new animal models through genetic engineering holds great promise. The mouse has distinct advantages for studies that translate to humans. It is a small, short-lived mammal with a fully sequenced genome in which all life stages can be accessed, and for which myriad tools are available for precisely experimentally manipulating its genome. Further, the large collection of inbred strains of mice and the controlled environment in which the animals live provides the ability to confirm phenotype observations and to systematically perturb environmental factors and genetic input to measure effects under defined conditions. Current international efforts to 'make a mutation' for every gene through mutagenesis [1] and genetic engineering [2,3] make it imperative for phenotype data to be represented in standard descriptive formats to enable computational analysis and comparison.

Mammalian phenotypes are frequently genetically complex. Mutation of even a single gene almost always produces pleiotropic effects. Conversely, non-allelic mutations can produce indistinguishable phenotypes. Modifier genes and epistatic interactions can markedly alter the phenotype. Combining different allelic combinations of different genes can produce unique phenotypes not found in the single-gene mutation genotype. Imprinting of genes can dramatically affect phenotype. Mutations expressed in different inbred strains of mice can manifest as an increase or decrease of severity or penetrance of the corresponding phenotype. Quantitative trait loci (QTL) can contribute in complex nonlinear ways to the phenotype. In addition, mutations that are 'genomic' in nature, either disrupting or deleting multiple genes or occurring in intergenic regions, can produce distinct phenotypes and challenge us to think beyond gene effects to genomic effects. The outcome of these complex interactions can be dissected and reproducibly examined by characterizing inbred strains that represent the combined phenotype of the 'whole-genome' genotype in its environmental context.

The Mouse Genome Database (MGD) at the Mouse Genome Informatics website [4,5] serves as the model organism database for mouse, representing the genetics, genomics and biology of the mouse and as a community resource for mammalian studies. Significant reorganization and modeling of phenotypes is now underway to support these data robustly, to represent phenotypes in ways that are computationally accessible, and to provide human interfaces to these data that will enable knowledge building and hypothesis generation. One component of this work is the development of the Mammalian Phenotype (MP) Ontology, a structured vocabulary that will aid in standardizing annotations and, with its concepts definitions, unambiguously describe phenotypic observations.

## Results and discussion

### The problems of text

Written descriptions of phenotypes in higher organisms reflect the complexity of the subject, the richness of language, and the phenomenal diversity that these data represent. While text descriptions are commonly used in publications describing phenotype, and have been the basis of electronically accessible phenotypic descriptions (for example, Online Mendelian Inheritance in Man (OMIM) [6] and the Mouse Locus Catalog (MLC) [7], text is unreliable for searching, either manually or computationally. From the user's perspective, even the best full-text search including Boolean operators will miss appropriate records (false negatives) and return unwanted records (false positives).

Consider the example in Table 1 where searches were done to find spontaneous mutations in which mice were entirely or partially devoid of hair/fur. To obtain a complete result, the user would need to use a number of search terms and synonyms. The wording within the text depends upon the author of the record and his/her particular word usage and editorial style. A minimum of four search terms is needed to recover the 27 relevant mutations displayed in this table and it cannot be ascertained if this is a complete set of mutations for this phenotype. Conversely, the user is returned with 23 irrelevant results. Irrelevant results can be returned for many reasons including, but not limited to, the following: the author of the record is contrasting the phenotype of a mutation in one gene with a mutation in another gene; the author is making a statement that includes the negation of the trait; the match is based on gene name rather than phenotype; the mutant was used as a linkage marker to genetically map another gene.

A further detriment to database text records is their difficulty to update and maintain. As new information is learned about a phenotypic mutant, the record must be continually rewritten. Although this practice might be sustained for a small number of records, it does not scale when thousands of mutant records are considered. The alternative of simply adding on another paragraph to existing text records becomes confusing, with potentially conflicting information and different writing styles appearing in one textual description, and unwieldy, with more and more text that may no longer represent a logical synthesis.

### Nomenclatures, vocabularies and ontologies

Formal nomenclatures for genes, mutant alleles and inbred strains of mice have existed since the 1940s [8,9]. The MGD [4] serves as the authoritative source for the names and symbols associated with mouse genes, alleles and strains. The advantage of applying such nomenclatures has been increasingly recognized as genomes become better defined and the realized power of comparative genomics allows homologous and orthologous gene relationships to be explicitly defined. At present, human, mouse and rat gene nomenclatures operate in parallel, using coordinated symbols for all three species' genes. In addition, mouse and rat strain nomenclatures were merged to one standard strain nomenclature recently, making strain identity and nomenclature conventions consistent. Nomenclature guides for mouse and rat genes, mutant alleles, and strains are available online and regularly revised based on international nomenclature committees' reviews [10].

Beyond nomenclatures, which are key to object identities and relationships, are vocabularies that can be used to describe broader concepts and categorizations. Vocabularies can take many forms, including simple lists of controlled terms, such as the cytogenetic band designations used to name the bands defined by chromosome staining or the classes of genetic markers, such as gene, pseudogene, expressed sequence tag (EST), and so forth.

The annotation of complex biological data and concepts requires more than lists and simple vocabularies. Ontologies, or 'descriptions of what there is', contain both concepts, with precise meanings, and relationships among those concepts. As such ontologies are able to support descriptions of complex biology and are useful in making these data more amenable to computational analyses. The first widely used ontology developed and adopted in the biological domain is the Gene Ontology (GO) [11-13] which contains concepts of molecular function, cellular localization and biological process for annotating the functional aspects of genes. The GO is structured as a directed acyclic graph (DAG), where each vocabulary term (node) may have both multiple parent term and multiple child term relationships. MGD uses GO extensively for gene annotation [14]. In addition, MGD has adopted the Mouse Embryo Anatomy Nomenclature Database [15] and the Anatomical Dictionary for the Adult Mouse [16] for annotating data that include anatomical attributes, such as tissue sources for clones and phenotypes. The Gene Expression Database (GXD) [17], integrated with MGD through the Mouse Genome Informatics (MGI) system [4], applies these anatomical ontologies as a central concept in the description of expression data.

### Mammalian Phenotype Ontology

Although the need for vocabularies as key components to consistent phenotype annotations for mammals has been recognized for some time [18], and many smaller controlled vocabularies have been implemented to describe various aspects of phenotype in MGD (for example, class of mutation, embryonic stem (ES) cell lines used for generating targeted mutations, type of inheritance), much of the data has remained in text form. Over the past two years, the Mammalian Phenotype (MP) Ontology has emerged to more precisely describe phenotypes, and to allow easier access to phenotype-sequence interactions.

Our goal is to describe the richness of phenotypes as precisely as they are known, recognizing that phenotype data are by nature complex and usually incomplete. Taking advantage of structural properties of a DAG, we have the ability to annotate phenotypes to the level of data resolution available, whether general or very specific and the ability to query with a high-level term, returning all phenotypes containing annotations to that term or to terms more specific than the query term. Thus, one can query for 'respiratory signs/symptoms' and retrieve all phenotypes annotated to this term and its hierarchical 'children' (abnormal breathing, abnormal respiratory sounds, anoxia, apnea, dyspnea, hypercapnia, and so on), or specifically request annotations to any of these sub-terms.

**Figure 1:**
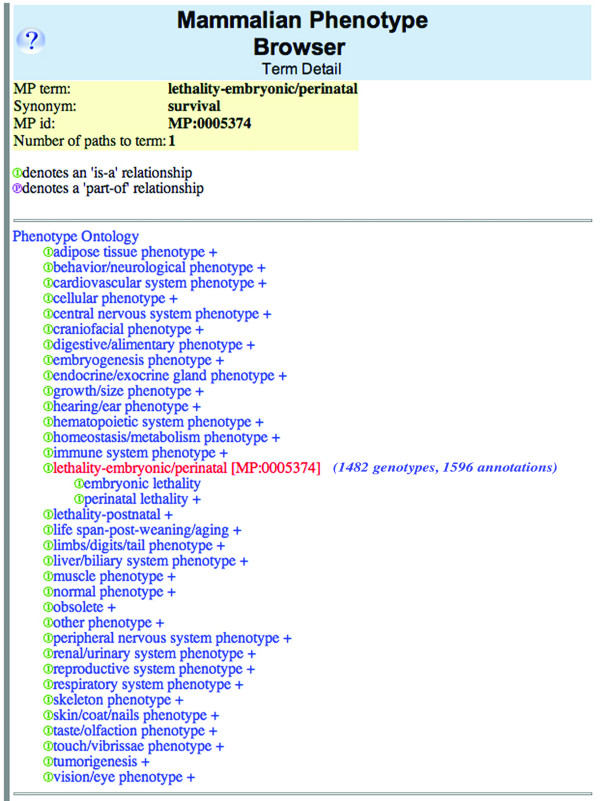
Screen shot of the Mammalian Phenotype (MP) Browser in which the term 'lethality-embryonic/perinatal' was selected. At the top (yellow shading) the term, its synonym and MP unique identifier appear. The number of paths to term (in this case, one) indicates how many paths through the DAG structure can be traversed to reach the term. The main body of the browser page shows the selected term highlighted and within the context of the hierarchical path(s) of the MP Ontology. In this example page, three levels of the hierarchy are visible (Phenotype Ontology, its 34 sub-terms, and the two sub-terms that fall beneath 'lethality-embryonic/perinatal'. The plus sign, appearing for many of the terms on this page, indicates that these terms have additional sub-terms that can be viewed by clicking on the term to expand the view of that portion of the ontology. The number of genotypes and annotations following the term 'lethality-embryonic/perinatal' is a hypertext link to those data. This latter feature will be available in early 2005.

The top level terms of the MP Ontology include physiological systems, behavior, developmental phenotypes and survival/aging. Physiological systems branch into morphological and physiological phenotypes at the level immediately below. A browser to view the ontology is available at [19] (Figure 1). In this browser the DAG structure is flattened into a hierarchy, with multiple hierarchies representing unique paths to a term displayed sequentially. MP terms and synonyms can be searched or users can browse the ontology starting from the high-level terms and open levels continuously to increasingly granular terms.

Each MP ontology term has a unique identifier, a definition and synonyms. In the term detail pages, these data and the number of hierarchical paths of the vocabulary where the terms appear are displayed. A plus sign following the term indicates that children of this term exist. In this figure, displayed next to the term, is a link indicating the number of annotation instances in MGD using this term or children of this term. This feature, due to be publicly available in early 2005, will greatly improve phenotype-centric searching in MGD.

### Developing the MP vocabulary

To initiate the vocabulary, we first developed a high-level categorization of phenotypes consisting of approximately 100 terms, such as heart/cardiovascular dysmorphology and skeletal axial defects. As we used this list for annotations, terms were refined and general organizing principles for the MP vocabulary were developed.

An important component of our approach has been to address two practical implementation questions. From the biologist's perspective, the question is what term would be used to describe a specific phenotypic trait. From the curation perspective, we ask what terms reflect biological reality and maximize curator productivity.

From a purely ontological perspective, every trait could be broken down into a core object, such as 'cornea' or 'gastrulation', defined by anatomical, behavioral or physiological terms, and a series of attribute vocabularies that describe the quality, quantity and character of a trait. For the practical reason of needing robust terms to describe phenotypes up-front to speed curation and the problem of losing biological meaning, particularly for clinical or dysmorphology terms, when terms are completely deconstructed (that is, the sum of the parts is less than the term itself), we have chosen to use compound terms in the MP Ontology. A few examples of terms where it is difficult to preserve the full biological meaning once they are deconstructed are shown in Table 2. In addition, it should be noted that each of these terms requires multiple annotations to recover all aspects that the single term provides. Use of complex terms in the MP Ontology, however, does not preclude also storing the decomposed version should this later prove desirable (see PATO model discussed in [20]). More important, the MP Ontology can currently hold, for each term, database cross-references to other ontologies. This is a common practice in GO when compound terms are developed. For the MP Ontology, these cross-references include anatomical terms from the Mouse Anatomy ontologies [15,16] and the GO process terms [21].

Three major strategies are being pursued to further develop the vocabulary itself. First and most important is through the ongoing process of curating phenotype data. As new phenotypic traits are described and published, the need for new terms is recognized. New terms added in this way may be a simple addition to an existing hierarchical path or may result in the addition of entire new branches in the hierarchy. Second, collaborative efforts between the MGD phenotype curators, the mouse mutagenesis centers and the rat genetics community identify new specific terms and suggest improved organization of terms within particular hierarchical branches. Third, we are recruiting individuals with expertise in specific biological domains to review and evaluate sections of the vocabulary for accuracy, completeness and systematic arrangement. The MP Ontology is a work in progress and remains incomplete in some areas. We welcome the participation of the mammalian research community so that the most useful, definitive and universally applicable terms will be included. Information can be obtained by sending e-mail to pheno@informatics.jax.org.

While common pathological and clinical terms are used in the MP Ontology, considerations for term placement within the structure and for precise terminology is often derived from comparison with other open biological ontologies (OBO) [22]. Recently, a cell-type ontology has become available [23] and a comparison of terminology to this ontology has not yet been completed. We are working with the mutant mouse pathology database Pathbase [24,25] to map and cross-reference terms from their Pathology Ontology.

### Vocabulary tools

The MP Ontology was built as a DAG using the DAG-Edit software written by John Richter and Suzanna Lewis [26]. The MP Ontology is updated daily and can be browsed or searched online at [19]. MP files also are available in flat file format and OBO format from our ftp site [27] and are posted at the OBO site [28].

### Phenotype data annotation

**Figure 2:**
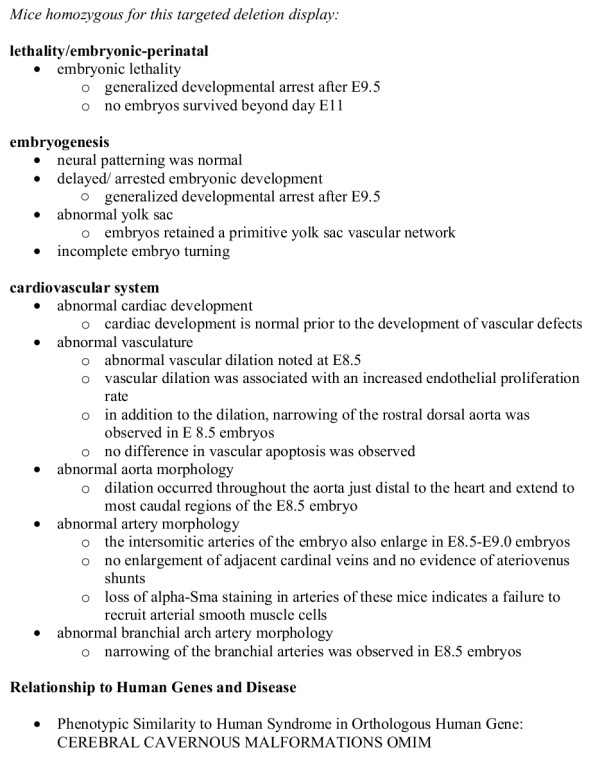
Example showing a portion of the MGI web display of the phenotype annotations for homozygotes for the *Ccm1*^*tm1Dmar *^genetically engineered allele (the first targeted mutation from the Douglas Marchuk laboratory in the cerebral cavernous malformations 1 gene). Homozygotes are embryonic lethal, showing developmental and cardiac abnormalities. Note the organization of annotations under the high level phenotype categories and the link to OMIM where the mouse and human show similar phenotypic characteristics. See [30] to view the complete record for the *Ccm1*^*tm1Dmar *^phenotypic allele. Searches for phenotypes at MGI can be done via the Alleles and Phenotypes Query Form [31].

Phenotypes are described in the MGD relative to the genotype of the individual. Genotype objects specifically consist of one or more allele pairs describing mutations or QTLs and the genetic background strain(s) where the phenotype was observed. Each phenotype annotation associates a MP Ontology term with a genotype/strain and the reference or data source supporting this assertion. Additional modifying text may be annotated to describe detail that is not easily standardized. Examples include experimental conditions, age of onset and incidence, and trait penetrance, among others. The annotation note may also include specifics of the phenotype where such details are deemed to be too case specific to be a MP term. In addition, genotypes are associated with OMIM where a particular mouse genotype is a model for human diseases and syndromes. Figure 2 shows the portion of one phenotype record that uses the MP Ontology.

## Conclusions

The MP Ontology and annotation schema was designed to minimize curatorial time, yet remain precise enough to describe phenotypic data. It supports robust phenotypic annotations and querying capabilities for mouse phenotype data. While this vocabulary is far from complete, we have designed strategies for its continued development as a collaborative effort for supporting the representation of existing mutations and those that continue to be created.

As of 1 November 2004, over 11,150 phenotypic alleles representing mutations in 5,214 unique genes had been catalogued in MGD. For these alleles, 9,696 genotype records exist, with 21,556 phenotypic annotation instances. The MP Ontology is also used in phenotypic data annotations at the RGD [29]. As our database groups continue to accumulate annotations, it will be possible to mine these data to ask interesting questions about similarities and differences in comparable allele effects between the species, as well as within species. Comparative phenotype data will potentially uncover new modifier effects and point to new pathway relationships and genetic networks tied to disease processes. The MP Ontology will be critical for enabling computational analyses and providing a framework for improved web views and other human-comprehensible displays for the research community.

## Figures and Tables

**Table 1:** Text search for mutations with hair/fur loss defects in the Mouse Locus Catalog

Hairless	Nude	Bald	Hair loss	Loss of hair	Furloss	Loss of fur	Composite 'true' result
			*Al*				*Al*
	Aldoc*						
					*alp*		*alp*
			*ao*				*ao*
*Bda*		*Bda*					*Bda*
	*Blai2*^†^						
	*Bmp6*^†^						
	*Btk*^†^						
			*Ctsl*				*Ctsl*
*dep*							*dep*
	*Dh*^†^						
		*Dsg3*	*Dsg3*				*Dsg3*
		*Ebp*					*Ebp*
*Eda*		*Eda*					*Eda*
		*Edar*					*Edar*
		*Edaradd*					*Edaradd*
			*exf*				*exf*
	*Fgf7*^†^						
	Foxj1*						
*Foxn1*	*Foxn1*		*Foxn1*				*Foxn1*
			*Frl*			*Frl*	*Frl*
			*hl*	*hl*			*hl*
	*Hoxb8*^†^						
*Hr*		*Hr*	*Hr*				*Hr*
	*Htr2b*^†^						
	*Il7*^†^						
	*Itgam*^†^						
	*Itgav*^†^						
			*jb*				*jb*
			*jd*	*jd*			*jd*
		*Krt2-6g*					*Krt2-6g*
		*ma*					*ma*
	*Ngef*^†^						
*Nras*^†^							
	*Ny*				*Ny*	*Ny*	*Ny*
*Otc*			*Otc*				*Otc*
		*Pdcd8*					*Pdcd8*
*Rbpsuh*^‡^							
*Rbpsuh-ps1*^‡^							
*Rbpsuh-ps2*^‡^							
*Rbpsuh-rs3*^‡^							
*Scd1*							*Scd1*
	*Shc1*^†^						
			*Slc30a4*				*Slc30a4*
*St*^§^							
			*tf*				*tf*
*Tgfb1*^†^							
	*Tgfbi*^†^						
	*Tnfrsf6*^†^						
*wal*							*wal*
15	18	10	14	2	2	2	27 true results 23 irrelevant

The term heading each column was used to search the Mouse Locus Catalog [7]. The gene symbols listed in the column are those returned in response to the search. Because this is a text search, any matching text within the written description of the phenotypes for mutants known for that gene will be counted as a match. The 23 irrelevant results were returned due to: *match based on mapping this gene close to the mutant displaying this phenotype; ^†^experimental result of using mice displaying this phenotype in relation to the gene in question; ^‡^match based on gene name, not phenotype; ^§^match based on another mutation which is stated as arising in a stock carrying this phenotype.

**Table 2:** MP Ontology terms and their decomposed forms

MP Ontology term*	Object	Body location (anatomy/cells)	Attribute	Modifier	Value
Hydrocephaly^†^	Cerebrospinal fluid	Brain cerebral ventricles	Amount	Relative	Excessive
	Brain		Size, mass		Increased
	Trauma	Brain	Qualitative		Observed
Dystrophic cardiac calcinosis^‡^	Calcium salts	Heart	Deposition		Observed
	Inflammation	Heart	Qualitative		Observed
	Lesions	Heart	Qualitative	Artherosclerotic	Observed
Lenticonus^§^	Eye lens capsule		Shape	Conical	Bulge
	Eye cortex		Shape	Conical	Bulge
Osteopetrosis^¶^	Trabecular bone		Amount	Dense	Excessive
	Cartilage		Amount	Calcified	Excessive
	Erythrocytes		Relative_number		Decreased
	Hematopoiesis		Location	Extramedullary	Ectopic

*Definitions from the MP Ontology. ^†^Hydrocephaly, excessive accumulation of cerebrospinal fluid in the brain, especially the cerebral ventricles, often leading to increased brain size and other brain trauma. ^‡^Dystrophic cardiac calcinosis, a condition characterized by the localized deposition of calcium salts in the heart; often occurring in association with inflammation or atherosclerotic lesions and other pathological states. ^§^Lenticonus, a conical bulging of the lens capsule and the underlying cortex of the eye. ^¶^Osteopetrosis, excessive formation of dense trabecular bone and excessive calcified cartilage formation; may lead to anemia and extramedullary hematopoiesis.
